# Mini Self-Retrieval Practices of Skeletal Muscles in the Human Gross Anatomy Course

**DOI:** 10.1007/s40670-024-02075-z

**Published:** 2024-09-28

**Authors:** Yuefeng Lu, Oheneba Boadum

**Affiliations:** Department of Advanced Biomedical Education, University of Mississippi Medical Center, 2500 North State St, Jackson, MS 39216 USA

**Keywords:** Self-retrieval practice, Skeletal muscles, Gross anatomy

## Abstract

Students in health professional studies are often faced with the daunting task of memorizing large volumes of information in a limited time. Gross anatomy is usually the first course students may take in their curriculum, and the volume and complexity of anatomical information students must memorize, including muscle names, become overwhelming. Here, we outline a teaching method and pedagogical application of self-retrieval in gross anatomy courses, where students were provided with a number system that served as goals and cues for easy retrieval of muscle names for different anatomical regions.

## Introduction

First-year dental and medical students are often overwhelmed by the requirement of memorizing and integrating complex morphological and functional terms from biochemistry, histology, and gross anatomy courses [1, 2]. Usually, remembering and retaining these vast quantities of requisite terms constitutes an overwhelming challenge for some students [3].

Retrieval practice is a more effective learning strategy than reviewing materials by re-reading [4, 5]. Even though it yields greater retention of factual and conceptual knowledge and strengthens memory, most students remain unaware of the power of retrieval practice [6, 7].

Our previous study on the cranial-nerve table practice showed how a single self-retrieval practice could significantly improve students’ performances [8, 9]. The positive student perception and improved academic outcomes from that study motivate us to continue using this method to facilitate students learning [8, 9].

As gross anatomy educators, we have long strived to deliver concise facts to students to lessen the extrinsic cognitive load and make learning easier. Where applicable, we have used a unique number and grouping system to help make complicated facts manageable for students’ learning and recall. Cues are effective in retrieving information from long-term memory and making them more accessible to working memory [10, 11]. Studies have shown that rehearsal prevents information decay during learning [12].

Over the past several years, we have used this numerical strategy to organize and teach skeletal muscles in each of the four anatomy blocks: the back and upper limb (block 1), thorax and abdomen (block 2), pelvis and lower limb (block 3), head and neck region (block 4).

This number system was developed to help students better recall muscles and retain muscle nomenclature in anatomy learning. Retrieval practice benefits learning and memory and is effective in learning complex materials [13]. Overt retrieval involving students saying or writing responses is associated with higher recall than covert recall or mentally answering questions and improves learning [13]. As such, the following number system was used as a cue to help students recall from memory muscles learned previously during the block to facilitate learning.

## Method

The dental/medical gross anatomy course at the University of Mississippi Medical Center (UMMC) is a dissection-based course that is divided into four blocks covering the following regions during each spring semester: back and upper limb (block 1), thorax and abdomen (block 2), pelvis and lower limb (block 3), and head and neck regions (block 4). A numerical system was developed to cue students to recall muscles in each region during their end-of-block review sessions to improve student retention and knowledge of muscle names, attachments, and actions. These reviews were conducted without prior notification and included in the last lecture at the end of each study block. It allowed students to engage faculty with questions and be guided through essential review materials. During these sessions, depending on which block was taught, students were asked to recall as many muscles in that specific region as possible, either by region or innervation to a region, e.g., list the muscles in the forearm or list the muscles supplied by the median nerve in this region. Students could collaborate with each other or work alone. After 2–3 min, the total number of muscles in that region or the muscles innervated by that nerve to the region was shared with the students without the muscle names. With the numbers provided, students reviewed their answers and, using that as a cue, tried to recall any other muscles they had missed. Where different regions were involved, e.g., the anterior compartment and posterior compartment of the thigh, these numerical cues were done by those compartments. Where applicable, the total number of muscles in each region was further divided into subregions using simple numerical strategies that served as additional cues for the students. Muscle groups for each region were shown to students on PowerPoints after their retrieval exercises. The various regions covered during the anatomy course by muscle numbers are as follows:

- Back and upper limb: 66 muscles
- Thorax and abdomen: 11 muscles
- Pelvis and lower limb: 60 muscles
- Head and neck: 72 muscles.

Below, the specific steps used for different blocks are outlined. Of note, the lengths of self-retrieval practices were varied in different blocks due to content variations.

### Block 1. Upper Limb and Back

This block has a total of 66 muscles. To make this block manageable, the 66 muscles were divided into 16 for the back and 50 for the upper limb. The five superficial back muscles were included in the upper limb group because of their common primary functions. Students were provided the information below for the back muscles after their initial recall.

#### Back Muscles (16 Muscles)

1. Intermediate back: 2 muscles
2. Deep back (10): 1 Spinotransversales muscle; 3 muscles for the erector spinae group; 3 Transversospinales muscles; 3 Segmental muscles.
3. Suboccipital: 4 muscles in the posterior neck.

#### Upper Limb Muscles (50 Muscles)

For the 50 upper limb muscles, we provided students with a number for each of the four subregions. These were further divided, as shown below, with other learning strategies like mnemonics, where possible.

1. Shoulder region: 15 muscles
  Anterior: 3 muscles
  Lateral: 2 muscles (one lateral to the humerus and one lateral to the thorax)
  Posterior:
    Superficial: 5 muscles, mnemonic cue: TLL-Rmi-Rma
      (TLL-Rmi-Rma: Trapezius, Latissimus dorsi, Levator scapulae, Rhomboid minor, and Rhomboid major)
    Deep: 5 muscles, mnemonic cue: SIS-Tmi-Tma
      (SIS-Tmi-Tma: Supraspinatus, Infraspinatus, Subscapularis, Teres minor, and Teres major)
2. Arm: 4 muscles
  Anterior: 3 muscles
  Posterior: 1 big muscle
3. Forearm: 20 muscles
  Anterior (8): 2 pronator, 2 flexor carpal, 2 longus, and 2 flexor digitorum
  Posterior (12): 3 for thumb, 3 for fingers, 3 for wrist, and 3 others, cued as ABS (ABS, for Anconeus, Brachioradialis, and Supinator)
4. Hand: 11 muscles
  Lateral (4): 3 thenar muscles plus 1 more for the thumb
  Intermediate: 3 muscles, all with multiple bellies
  Medial (4): 3 hypothenar muscles plus 1 more in the superficial fascia

### Block 2: Thorax and Abdomen

This block has 11 muscles, and the following cues were provided after students’ initial recall.

- Thorax (5): anterior 1, lateral 3, and posterior 1
- Abdomen (5): anterior 1, lateral 3, and posterior 1
- Additional cue used for the diaphragm: one muscle separates these two cavities

### Block 3: Pelvis and Lower Limb

There are 60 muscles in this block. These were divided broadly into lower limb (50) and pelvis (10). For easier recall, these muscles were further divided into subregions and cued using the following numbers from 10 to 14: pelvis (10), hip (11), foot (12), leg (13), and thigh (14).

1. Pelvis: 10 muscles cued as 1, 2, 3, 4, as shown below.
  1 muscle in the anal triangle
  2 muscles in the pelvic diaphragm
  3 muscles in the superficial pouch of the urogenital triangle
  4 muscles in the deep pouch of the female urogenital triangle (male has only 2)
2. Hip: 11 muscles
  Anterior hip: 2 muscles
  Posterior hip: 9 muscles (cued as 3, 3, 3 as follows: 3 Gluteal muscles; 3 muscles combine and share the common insertion (obturator sandwich)); 3 others (PTQ, i.e., Piriformis, Tensor fascia lata, and Quadratus femoris)
3. Foot: 12 muscles
  Dorsal: 2 muscles
  Sole: 10 muscles in four layers (from superficial to deep layer):
    Layer 1: 3 muscles — cued further as AFA for Abductor hallucis, Flexor digitorium brevis, and Abductor digiti minimi
    Layer 2: 2 muscles — Quadratus plantae, Lumbricals
    Layer 3: 3 muscles — cued further as FAF, for Flexor hallucis brevis, Adductor hallucis, and Flexor digiti minimi
    Layer 4: 2 interossei muscles — Plantar interossei, Dorsal interossei
4. Leg: 13 muscles
  Lateral: 2 muscles
  Anterior: 4 muscles — cued as “Tom, Henry, Dick and Fib” (for Tibialis anterior, Extensor hallucis longus, Extensor digitorum longus, and Fibularis tertius — medial to lateral)
  Posterior: 7 muscles
    Superficial triad: 3 muscles cued as GPS (for Gastrocnemius, Plantaris, and Soleus)
    Deep: 3 muscles — cued as “Dick, Tom, and Henry” (for Flexor Digitorum longus, Tibialis posterior, and Flexor Hallucis longus — medial to lateral).
    Behind the knee: 1 muscle to unlock (pop) the knee (Popliteus)
5. Thigh: 14 muscles
  Posterior: 3 muscles share a common origin
  Anterior: 5 muscles — Quadriceps and the longest muscle
  Medial: 6 muscles — 3 adductors and 3 as GOP (GOP: Gracilis, Obturator externus, and Pectineus)

### Block 4: Head and Neck

There are 72 muscles in this block. The muscles innervated by cranial nerves (CN) were introduced to the class in a previous review via a cranial-nerve table, and the total number was 60 [8, 9]. Cervical spinal nerves innervate the remaining 12 muscles. These are presented here.

1. CN GSE (General somatic efferent): 16 muscles
  CN III, IV, and VI together: All 7 extraocular muscles
  CN XI: 2 muscles
  CN XII: 7 muscles — All 4 intrinsic and 3 of the 4 extrinsic tongue muscles
2. CN SVE (Special visceral efferent): 44 muscles
  CN V: 8 muscles — 4 mastication muscles, 2 tensors, and 2 others innervated by mylohyoid branch
  CN VII: 22 muscles — 19 facial expression muscles and 3 others, including the smallest muscle
  CN IX: 1 muscle
  CN X: 13 muscles
    Soft palate: 4 of the 5 soft palate muscles, except for the tensor
    Pharynx: 4 of the 6 pharyngeal muscles except for the one innervated by CN IX and the one belonging to the soft palate
    Larynx: 5 of the laryngeal muscles: one for high pitch and one for low pitch; one to open and two to close
3. Neck muscles innervated by cervical spinal nerves: 12 muscles
  2 rectus capitis muscles
  2 longus muscles
  3 scalene muscles
  5 hyoid muscles: 1 suprahyoid and 4 infrahyoid

## Results and Discussion

Presenting these numbers per anatomical region served as cues for students to practice recalling learned materials. Because students struggled to remember all muscles, numbers per region were used to visually cue them into recalling missing muscle names. While students may not necessarily need to know every muscle in the body, in the first preclinical year, many gross anatomy questions at our institution are lower-level Bloom’s taxonomy-based questions that involve retention and recall of information [14]. These end-of-block reviews served two purposes: using numbers as cues to facilitate students’ recall of information from long-term memory and the overt retrieval and student discussions enhancing the effectiveness. During these sessions, students often appeared content, being able to remember muscles they had forgotten after cues were provided.

A critical instructional implication in the cognitive load theory involves not overloading students’ working memory with too much material [15]. Considering the four anatomy blocks had 208 muscles in total that students had to be conversant with, we developed this number and grouping system to lessen students’ burden in learning the material. However, counting the trapezius twice in the two blocks — Upper limb and back; Head and neck regions — gives a total number of 209 muscles. Large numbers of muscles, typically difficult to recall and complicated to teach, were organized into groups using simple number patterns and presented to students as cues to facilitate their recall. For example, recalling most of the 60 muscles in the lower limb was arduous for most students, even at the recall and identification level. However, when we presented this content through groupings and numbers, students who struggled to recall the muscles could remember more than they did. By using the number sequence 10, 11, 12, 13, and 14 to represent the muscles in these regions: pelvis (10), hip (11), foot (12), leg (13), and thigh (14), students were able to recall most muscles per region.

Similarly, when asked to recall muscles in the thorax and abdomen (block 2), some students struggled with it. Muscles in the thorax and abdomen were cued using the number five (5), and the common embryological origins of these muscles (hypaxial myotomes) were highlighted as it contributed to the similarities between them [16]: the five muscles in the thorax and abdomen were presented to students strategically as: 1 anterior, 3 lateral, and 1 posterior muscle in each region, separated by 1 diaphragmatic muscle. This allowed students to recall the transversus thoracis and the rectus abdominis muscles — the anterior muscles in the thorax and abdomen. Being prompted that the lateral muscles in each of these regions were three in number helped students recall the intercostal muscles (external, internal, and innermost — thorax), the external and internal obliques, and the transversus abdominis muscles for the abdomen. These cueing methods align with previous research on activation information in long-term memory through cues and memory and learning [17–19].

Another pattern that helped students was with the muscles of the forearm. After students attempted to retrieve the muscles from memory, we gave them this cue: eight anterior muscles in pairs and 12 posterior muscles in threes. This enabled students to retrieve the names of paired muscles in the anterior compartment, e.g., pronator teres and pronator quadratus. At this point, what they were trying to retrieve became more apparent to students.

Similar numbers were used to make recalling the muscles easier for students, who expressed the desire for more teaching strategies to facilitate their learning. Additional cues and mnemonics were sometimes used in some regions (e.g., the shoulder region). By doing this, we sought to replicate pre-existing theories of the associative network memory model and principles of encoding-retrieval specificity that utilized cue distinctiveness [19, 20]. These authors showed that using self-generated cues and mnemonics facilitated the spreading activation nature of memory and presented learners with an opportunity to capitalize upon the distinctiveness of the cue [19, 20].

The key was not only how many muscles students could recall but also providing an opportunity for enhanced learning and retention through self-retrieval exercises. Students were encouraged to pay more attention to the benefit of the retrieval process and how it allowed them to better organize and remember facts for a better recall next time. This has been shown to enhance propositional networks in long-term memory and enable students to retrieve the correct information more easily [21]. Pearson and Wilbiks [10], reporting on the effects of audiovisual memory cues on working memory, stated that cues yielded significantly higher recall. Further, in line with existing literature about the successful recall of information from memory depending on the provision of retrieval cues, these review activities saw students recalling more muscles following cueing [22]. Even when students missed the correct muscle name, the to-and-from actions of effortful retrieval, in line with existing research, should strengthen memory and facilitate learning [22]. In addition, unprepared students could benefit from these self-retrieval practices if the correct information is immediately provided after inconclusive recall [4, 22]. Together, advantages made the task manageable with less uncertainty, so students had a goal to reach, guided by the number of muscles. Thus, these mini reviews were conducted without any prior notification.

## Conclusion

The self-retrieval approach as a suitable learning method was practiced and emphasized at the end of each block. Students affirmed that they found it helpful and applicable in other courses. They commented that these quick reviews allowed them to recall more muscles learned earlier and hoped we could provide similar practices in other systems.

Recent advances in educational research have greatly enhanced our understanding of how the human brain acquires and retains knowledge [23]. While a formal assessment was not conducted among our students, students’ recall of information and retrieval of muscle names, including those innervated by specific nerves in a region, were better than before cues were given. Self-retrieval method, self-generated cues, and mnemonics were further emphasized as a good way of recalling and enforcing encoded knowledge, memorizing, and organizing large quantities of terms as often encountered in their health professional’s education.

Our mini self-retrieval practices have the potential to help students more efficiently recall, encode, and retain otherwise fragmented knowledge of overwhelming medical terms. Using the practices described here, students could individually and collaboratively test each other using self-retrieval practices and develop their variations of self-retrieval methods to facilitate their learning process. We look forward to further applying similar strategies in other courses as we explore other opportunities to provide self-retrieval practices to students.
